# A risk identification model for detection of patients at risk of antidepressant discontinuation

**DOI:** 10.3389/frai.2023.1229609

**Published:** 2023-08-24

**Authors:** Ali Zolnour, Christina E. Eldredge, Anthony Faiola, Yadollah Yaghoobzadeh, Masoud Khani, Doreen Foy, Maxim Topaz, Hadi Kharrazi, Kin Wah Fung, Paul Fontelo, Anahita Davoudi, Azade Tabaie, Scott A. Breitinger, Tyler S. Oesterle, Masoud Rouhizadeh, Zahra Zonnor, Hans Moen, Timothy B. Patrick, Maryam Zolnoori

**Affiliations:** ^1^School of Electrical and Computer Engineering, University of Tehran, Tehran, Iran; ^2^School of Information, University of South Florida, Tampa, FL, United States; ^3^College of Health Sciences, University of Kentucky, Lexington, KY, United States; ^4^Biomedical and Health Informatics, University of Wisconsin-Milwaukee, Milwaukee, WI, United States; ^5^School of Pharmacy, University of Pittsburgh, Pittsburgh, PA, United States; ^6^School of Nursing and Data Science Institute, Columbia University, New York, NY, United States; ^7^Center for Home Care Policy and Research, VNS Health, New York, NY, United States; ^8^Department of Health Policy and Management, Johns Hopkins University, Baltimore, MD, United States; ^9^Lister Hill National Center for Biomedical Communications, National Library of Medicine, National Institutes of Health, Bethesda, MD, United States; ^10^Center of Biostatistics, Informatics, and Data Science, MedStar Health Research Institute, Washington, DC, United States; ^11^Department of Psychiatry and Psychology, Mayo Clinic, Rochester, MN, United States; ^12^Collage of Pharmacy, University of Florida, Gainesville, FL, United States; ^13^Department of Biomechanics, Bu-Ali Sina University, Hamedan, Iran; ^14^Department of Computer Science, Aalto University, Otaniemi, Finland

**Keywords:** antidepressant discontinuation, adverse drug events, antidepressant effectiveness, online healthcare forums, content analysis, machine learning

## Abstract

**Purpose:**

Between 30 and 68% of patients prematurely discontinue their antidepressant treatment, posing significant risks to patient safety and healthcare outcomes. Online healthcare forums have the potential to offer a rich and unique source of data, revealing dimensions of antidepressant discontinuation that may not be captured by conventional data sources.

**Methods:**

We analyzed 891 patient narratives from the online healthcare forum, “askapatient.com,” utilizing content analysis to create PsyRisk—a corpus highlighting the risk factors associated with antidepressant discontinuation. Leveraging PsyRisk, alongside PsyTAR [a publicly available corpus of adverse drug reactions (ADRs) related to antidepressants], we developed a machine learning-driven algorithm for proactive identification of patients at risk of abrupt antidepressant discontinuation.

**Results:**

From the analyzed 891 patients, 232 reported antidepressant discontinuation. Among these patients, 92% experienced ADRs, and 72% found these reactions distressful, negatively affecting their daily activities. Approximately 26% of patients perceived the antidepressants as ineffective. Most reported ADRs were physiological (61%, 411/673), followed by cognitive (30%, 197/673), and psychological (28%, 188/673) ADRs. In our study, we employed a nested cross-validation strategy with an outer 5-fold cross-validation for model selection, and an inner 5-fold cross-validation for hyperparameter tuning. The performance of our risk identification algorithm, as assessed through this robust validation technique, yielded an AUC-ROC of 90.77 and an F1-score of 83.33. The most significant contributors to abrupt discontinuation were high perceived distress from ADRs and perceived ineffectiveness of the antidepressants.

**Conclusion:**

The risk factors identified and the risk identification algorithm developed in this study have substantial potential for clinical application. They could assist healthcare professionals in identifying and managing patients with depression who are at risk of prematurely discontinuing their antidepressant treatment.

## 1. Introduction

Antidepressant use among U.S. adults increased from 7.7% in 1999–2002 to 13.2% in 2015–2018, and the global market cost may reach $15.8 million by 2023. However, medications used to treat depression may require weeks to achieve an adequate response and patients' varied responses to depression treatment often require medication adjustment (Ogle and Akkerman, 2013). Additionally, adverse events can lead to discontinuation, especially with selective serotonin reuptake inhibitors (SSRIs) and serotonin-norepinephrine reuptake inhibitors (SNRIs), causing withdrawal symptoms, depression relapse, emergency visits, and added strain on the patient, caregivers, and healthcare system (Lejoyeux and Adès, 1997; Zolnoori et al., 2018; Fava and Cosci, 2019). Hence, studies recommend tapering these medications over several months to minimize withdrawal (Horowitz and Taylor, 2019).

Despite evidence of the effectiveness of long-term use of antidepressants, it's common for patients to discontinue antidepressant therapy, a trend that varies over time (Vinkers et al., 2021). Discontinuation within the first 30 days of therapy is observed in up to half of the patients, a rate that escalates with each passing month (Olfson et al., 2006). Certain groups, such as those over 60 years, have been shown in studies to have a high non-adherence rate (Holvast et al., 2019). Other research has identified several factors linked to patients stopping their antidepressant medication(s), including ethnicity, socioeconomic status, perceived effectiveness, and experienced adverse effects during therapy (Olfson et al., 2006; Kales et al., 2016; Fava and Cosci, 2019). A study in Sweden over a 2 year period identified low socioeconomic status as a risk factor of non-adherence (Kales et al., 2016).

Furthermore, several studies on patient non-adherence to antidepressant medications have identified adverse drug reactions/events (ADRs) as a leading factor, including both qualitative and quantitative studies (Ho et al., 2017). In addition to patient experiences with ADRs, clinical studies have shown the correlation and importance of patient beliefs in antidepressant medication discontinuation (Aikens et al., 2008). In a Malaysian study of 30 patients in a psychiatric government run clinic, most patients in the study believed antidepressants were harmful (Aikens et al., 2008). There is need for further study of the factors which lead to medication non-adherence in order to improve patient management of this disease and tailor patient education on antidepressant medication to address these beliefs (Anderson and Roy, 2013; Srimongkon et al., 2018). A small semi-structured interview study (*n* = 23) of consumer-related factors in antidepressant non-adherence in Sydney, Australia revealed beliefs and experiences are important in assessing risk for non-adherence. Therefore, this study aims to further analyze patient perceptions of their experiences with antidepressant medication adverse events, effectiveness, patient-provider communication, and perceived knowledge on the likelihood of medication adherence from a consumer health perspective (both positive and negative aspects), as most of these above prior studies are the context of clinical and hospital settings or specific to a particular geographic setting. In this study, we aimed to delve deeper into the factors influencing antidepressant discontinuation by analyzing patients' self-reported experiences on online healthcare forums.

The literature acknowledges previous research of free text data concerning psychiatric medications, derived from hospital-based datasets (Iqbal et al., 2017). Studies have delved into unstructured free text data from electronic health records (EHRs) in UK-based psychiatric hospitals to extract adverse drug events related to antipsychotics and antidepressant medications (Iqbal et al., 2017). One particular study, referred to as the “Adverse Drug Event Annotation Pipeline (ADEPt),” analyzed a collection of psychiatric clinical notes. They employed a rule-based natural language processing method to examine adverse events tied to discontinuation of psychiatric medication. Furthermore, the Mental Health Case Register, utilizing the GATE NLP software tool, extracted adverse events associated with antidepressant discontinuation.

Social media platforms like askapatient.com are frequently used to document patient experiences with treatments like antidepressants. Past studies highlighted its usefulness as a data source supplementing the FDA's Adverse Event Reporting System for pharmacovigilance. Recently, researchers are using social media to investigate factors tied to adverse outcomes (Zhou and Hultgren, 2020; Lee et al., 2021). Due to the large volume of social media data, natural language processing (NLP) and machine learning (ML) have become ideal for automatic analysis of patients' narrative reports. However, performance of these automated methods can be compromised by colloquial language and ambiguous terms in patient reports. Prior research found that rule-based text mining systems often struggle to extract self-reported antidepressant side effects from social media (Zolnoori et al., 2019a,b,c). Also, machine learning-based NLP systems often underperform due to a limited annotated corpus of patient-narrative data (Sarker and Gonzalez, 2015). These systems use annotated data to learn patterns, make predictions on known outcomes, and apply these inferences to unseen data (Sarker et al., 2015; Zolnoori, 2017).

This study aimed to create an annotated sample (corpus) of risk factors contributing to antidepressant non-adherence, using patient self-reported narrative data in online healthcare communities (social media forums). This online community perspective will add to existing literature on patient perceptions and medication non-adherence as discussed above. We demonstrated the usability of this corpus by formulating a risk identification algorithm designed to proactively identify patients at risk of non-adherence with their antidepressant regimen.

## 2. Methods

**Figure 1:**
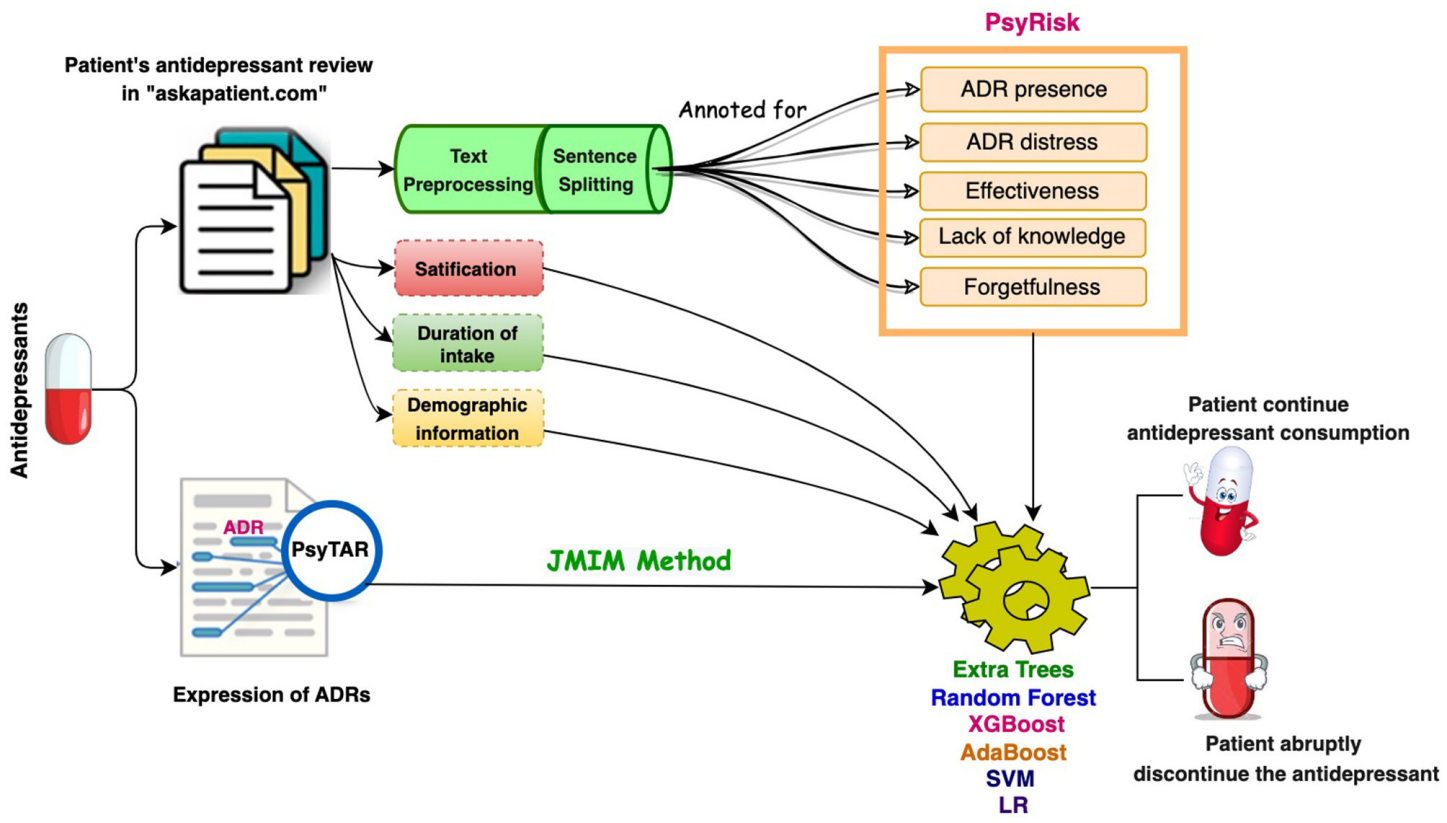
A schematic view of the methodology of the study.

Please see Figure 1 for a schematic view of the methodology of the study.

### 2.1. Data source

This study relies on data from a healthcare forum, “askapatient.com,” a platform dedicated to the collection of patients' self-reported experiences with a variety of medications. The forum organizes the collected data in a tabular format, comprising eight fields: (1) patient satisfaction rating [on a scale from 1 (not satisfied) to 5 (strongly satisfied)], (2) reason for prescription, (3) adverse drug reactions (ADRs), (4) patients' comments, (5) gender, (6) age, (7) duration of medication intake, and (8) date of comment submission. While patients are specifically encouraged to report ADRs, providing information on other aspects of medication, such as its effectiveness or their prior knowledge about it, is optional. All data on this forum is anonymous and publicly accessible. For the structure of the data in this forum, refer to Appendix A. As the study data are publicly available, the University of Wisconsin-Milwaukee's institutional review board considered patient consent unnecessary and exempted this study.

### 2.2. Antidepressants sources

We collected data for four prevalent antidepressant medications: Zoloft (Sertraline), Lexapro (Escitalopram) from the SSRI class, and Effexor XR (Venlafaxine), Cymbalta (Duloxetine) from the SNRI class. According to the National Institutes of Health (NIH) MedlinePlus, these antidepressants are among the most prevalent ones prescribed in the United States (NIH MedlinePlus Magazine, 2021). The sample sizes for Zoloft, Lexapro, Cymbalta, and Effexor XR were 213, 219, 231, and 228, respectively. Details on creating the sample size (Charan and Biswas, 2013) for this study can be found in Appendix B.

### 2.3. Creating an analytical framework for data annotation

In this study, we employed the Framework Method with a deductive-inductive approach, a method previously developed by our team, to identify risk factors associated with antidepressant discontinuation (Ma and Eldredge, 2017; Zolnoori et al., 2018). Deductive and inductive methods, used for theme generation in content analysis, vary in their approach. The deductive method identifies themes via external sources, such as a literature review, while the inductive method identifies themes directly from narrative data using the open coding technique (Zolnoori et al., 2019c). During the deductive phase, we carried out an extensive literature review to summarize the factors associated with antidepressant discontinuation. Risk factors included poor tolerability of adverse effects (perceived distress from ADR) and lack of treatment response (antidepressant ineffectiveness). A comprehensive list of risk factors can be found in the previous study (Zolnoori et al., 2019c). Using these risk factors, we created an initial analytical framework to analyze the patients' reviews of antidepressants (van Servellen et al., 2011; Ho et al., 2017; Falcaro et al., 2019; Henssler et al., 2019).

During the inductive phases, we annotated roughly 30% (310) of the total 891 antidepressant reviews, chosen randomly, using the initial analytical framework created in the deductive phase. Given that each review encapsulates diverse aspects of medication, we segmented the subsample of reviews into sentences to aid the annotation process. To uphold the quality of data annotation, two health science background annotators independently annotated sentences for the presence or absence of risk factors. Any antidepressant review passages not captured by the initial analytical framework were discussed in team meetings to generate new themes. The inter-annotator agreement (IAA) calculated between annotators using Cohen Kappa was 0.72, indicating substantial agreement. In the final step of the inductive phase, risk factors appearing in fewer than 5% of the antidepressant reviews (e.g., lack of caregiver support) were either removed or merged with other risk factors. For example, Table 1 provides a list of the final risk factors, their descriptions, and examples of antidepressant reviews used to formulate the final analytical framework for annotating the entire study sample.

**Table 1:** List of the final risk factors, description, and examples of antidepressant reviews used for building the analytical framework.

**Risk factors**	**Description**	**Example from the patient's review**
**ADR presence**		
ADR presence	If the patient explicitly reported experiencing ADRs with antidepressants, the patient's post was marked as “1.”	“Effexor XR gave me horrible nightmares, and I kept waking up.”
ADR absence	If the patient explicitly reported no experience of ADRs, the patient's post was marked as “0.”	“I experienced no side effects.”
**Perceived distress from ADR**		
Perceived distress from ADR-high	If the patient explicitly mentioned that they suffered from ADRs, used any qualifiers indicating severity, or if the patient experienced limitations in daily functioning and social participation.	“The vertigo is rendering me unable to function in daily life, causing me to lose work.”
Perceived distress from ADR-medium	If the patient did not provide any information about the level of severity of the ADRs, we assume that the level of severity is medium.	“I didn't feel as lethargic yesterday but didn't go to sleep until 3 am.”
Perceived distress from ADR-low	Perceived distress from ADRs-low	“I do not think Lexapro gave me any serious adverse side effects.”
**Effectiveness**		
Effectiveness	If the patient explicitly mentioned that their depression symptoms improved or resolved after using the medication.	“It was brilliant for 2 weeks, except for some queasiness.”
Partial Effectiveness	If the patient explicitly mentioned that their depression symptoms improved or resolved after using the medication, but this improvement lasted only for a certain time period.	“This medication worked great only for 1 month. After that, my depression symptoms came back.”
Ineffectiveness	If the patient explicitly mentioned that their depression symptoms did not improve after using the medication.	“I haven't noticed any positive changes. No improvement in my symptoms.”
Patient's lack of knowledge about antidepressant	If the patient reports that they did not receive sufficient information about potential ADRs, the mechanism of the ADRs management, and their expectation about the medication's effectiveness.	“I had no clue it was the side effect from the Lexapro.”
Patient forgetfulness to intake antidepressant	If the patient explicitly reported that they forgot to take the antidepressant on time (missing a dosage) or ran out of medication.	“I missed a dose for 1–2 days, it really caused some problems (dizziness and nausea)”
^*^ **Patient-physician communication**		
^*^Patient-physician communication-positive	If the patient reported, he/she was satisfied with healthcare services provided by the clinician.	“Staying in touch with my physician helps me to be successful with the medication.”
^*^Patient-physician communication-negative	If the patient reported, he/she was not satisfied with healthcare services provided by the clinician.	“It seems that my doctor does not understand the crazy side effects of starting this class of drugs.”
Antidepressant discontinuation (outcome variable)	If the patient explicitly reported that they discontinued the antidepressant or they are in the process of discontinuation the antidepressant (weaning off or tapering off).	“Stopped the drug after 2 days and next day was feeling better.”

* This variable was removed from the list of predictor variables (for building the risk identification model) due to the low inter-annotator agreement and low frequency in patients' antidepressants reviews.

Out of a total of 6,009 sentences, the dataset exhibited 55 instances of positive patient-physician communication and 87 instances of negative patient-physician communication. However, these variables demonstrated a moderate IAA of 50% and a relatively low frequency within the patient reviews on antidepressants. Given these characteristics, we opted to exclude them from the list of predictor variables used in constructing our risk identification model.

### 2.4. Annotating the entire sample using the analytical framework

We applied the analytical framework to annotate the entire sample of antidepressant reviews (refer to Table 1). Similar to the inductive phase of annotation (see Section 2.3), we established the unit of analysis at the sentence level. The narrative reviews (891 antidepressant reviews) were segmented into sentences, yielding a total of 6,009 sentences. The same annotators involved in the prior study phase (Section 2.3) annotated all sentences using the risk factors outlined in the analytical framework (see Table 1). The total inter-annotator agreement (IAA) was 0.75, with the highest IAA for annotating “presence of ADR” (IAA = 0.87), and the lowest for annotating “patient satisfaction with patient-clinician interaction” (IAA = 0.5). Due to the low agreement between annotators and infrequent patient reports, “patient satisfaction with patient-clinician interaction” was excluded from the final annotation guidelines. This annotation phase resulted in a corpus of risk factors related to antidepressant discontinuation, offering potential for the creation of a risk identification algorithm for patients at risk of discontinuing antidepressants. This corpus, known as PsyRisk dataset.

Every risk factor was examined and annotated at the sentence level, but the presence of these risk factors was aggregated at the patient level. For instance, if three out of five sentences in a patient's antidepressant review were annotated for the presence of ADR, the patient-level aggregation would simply indicate the “presence of ADR.” The “PsyRisk” and the “Aggregated PsyRisk” datasets can be accessed publicly via the following link.

https://github.com/zonour97/Antidepressant_discontinuation/tree/main. Please see the annotated dataset in the “Datasets” folder.

### 2.5. PsyTAR corpus: annotated and normalized ADR expressions in patient's antidepressant review

For this study, we annotated patients' articulations of adverse drug reactions (ADRs), given the importance of ADR expression types in relation to antidepressant discontinuation. The methodology for this annotation was detailed in our prior study (Ho et al., 2017). The annotated ADRs form part of the PsyTAR corpus, encompassing ADRs, withdrawal symptoms, signs/symptoms, and diseases/disorders reported by patients with depression in their antidepressant review. All ADRs were standardized via mapping to the Unified Medical Language System (UMLS), a compendium comprising numerous controlled vocabularies within the biomedical sciences (Sarker et al., 2015). In total, the PsyTAR corpus incorporates 3,120 unique patient expressions of ADRs, normalized through mapping to 673 UMLS concepts. The identified ADRs were further classified into physiological (unique expressions of physiological ADRs = 2,048, normalized physiological ADRs = 411), psychological (unique expressions of psychological ADRs = 795, normalized psychological ADRs = 188), cognitive (unique expressions of cognitive ADRs = 197, normalized cognitive ADRs = 46), and functional categories (unique expressions of functional ADRs = 80, normalized functional ADRs = 28).

### 2.6. Development of a risk identification algorithm for the proactive identification of patients at risk of antidepressant discontinuation

To automatically identify patients at risk of discontinuing their antidepressant medication, we constructed a binary machine learning risk identification model using the PsyRisk and PsyTAR corpora.

#### 2.6.1. Identifying the most informative ADRs associated with antidepressant discontinuation

The expression of ADRs associated with antidepressants is one of the risk factors utilized in constructing the risk identification model. In total, the sample encompassed 673 standardized ADRs (as outlined in the Section 2.5) reported by patients in their reviews of antidepressants. Each ADR expression was considered as a binary variable with a value of “1” (if the patient reported the ADR) or “0” (if the patient did not report the ADR). Including all these variables in the ML models could heighten the risk of overfitting, thus compromising the model's generalizability to unseen data. To tackle this challenge, we selected the most informative ADR expressions using the Joint Mutual Information Maximization (JMIM) (Bennasar et al., 2015) method to construct the ML models.

The JMIM method accounts for potential dependencies among the feature set F = {f1, f2, …, fN} by choosing a subset of features S of dimension K, where K (the number of features in S) is <N (the number of features in F) and S is a subset of F. This subset, S, comprises features that minimize information redundancy among the selected features and maximize the joint mutual information between the feature set and outcome class (Y). The key advantage of JMIM over other feature selection methods, such as wrapper and embedding methods, is the generalizability of selected features, thereby enhancing stability and the ML models' generalizability to unseen datasets. More details about the JMIM method are provided in Appendix C.

#### 2.6.2. List of predictors variables and the outcome variable

For constructing the risk identification model, we structured the predictor variables into three components: (i) perceived qualitative risk factors annotated in the PsyRisk dataset as per Table 1, which include ADR presence, perceived distress from ADR (ADR distress), antidepressant effectiveness, patient's lack of knowledge about the antidepressant (lack of knowledge), and patient's forgetfulness to take the antidepressant (forgetfulness). Furthermore, “duration of intake” and satisfaction with the antidepressant (satisfaction) were part of the structured data collected by the “askapatinet.com” forum; (ii) demographic information such as age and gender; and (iii) type of reported ADR from the PsyTAR corpus (refer to Section 2.5). The outcome variable is the explicit patient report of “antidepressant discontinuation” (see Table 1 for definition).

#### 2.6.3. Handling missing values

To further refine the aggregated PsyRisk corpus for the development of the risk identification model, we imputed missing values for the predictor variables. For the risk factors in category (i), as outlined in Section 2.6.1, the annotation process focused on the presence or absence of the risk factor in the patient's narrative report. However, for the variable “effectiveness”—annotated for effectiveness, partial effectiveness, and ineffectiveness—this variable was marked as missing if no information was provided in the patient's narrative report. We imputed the missing value for this variable under the “missing at random” assumption, suggesting that the missing values can be imputed as a function of other predictor variables. We addressed the imputation by developing ML models using variables in the “perceived qualitative risk factors and duration of intake” category along with demographic information. Details of the ML development and evaluation for imputation are available in Appendix D.

For the variables age, gender, and duration of intake, we imputed missing values based on the assumption of them being missing completely at random, suggesting that the missing values are a random subset of the complete data. The variable “age” was imputed using the mean, “gender” was replaced by the mode, and “duration of intake” was substituted with the median.

#### 2.6.4. Machine learning algorithms used to develop the risk identification model

We employed various discriminative machine learning (ML) algorithms. These included Logistic Regression (Cokluk, 2010), which served as the baseline algorithm, Bootstrap Aggregation (Bagging) (Sun and Pfahringer, 2011), and Gradient Boosting ensemble decision trees (Freund and Schapire, 1996) as non-parametric ML methods with the capacity to generate a substantial number of decision trees (weak learners). Additionally, we used the Support Vector Machine (SVM) (Ben-Hur and Weston, 2010; Murty and Raghava, 2016; Wang et al., 2020), a parametric ML algorithm with the ability to employ both linear and non-linear kernels.

In the bagging decision tree methods we used, weak learners are trained independently, using equal weights for the final outcome. We applied popular Bagging algorithms, Random Forest and Extra Trees (Geurts et al., 2006). However, Gradient Boosting decision tree (Natekin and Knoll, 2013) methods like Adaptive Boosting (AdaBoost) and Extreme Gradient Boosting (XGBoost) (Chen and Guestrin, 2016) generate weak learners sequentially, accounting for previous errors. More details are in Appendix E.

#### 2.6.5. Evaluating the performance of ML algorithms

To evaluate the performance of the ML classifiers, we used a nested cross-validation strategy due to the relatively small size of our dataset. This approach entailed an outer 5-fold cross-validation for model evaluation and an inner 5-fold cross-validation for hyperparameter tuning. We employed stratified cross-validation in both loops to enhance the generalizability of the risk identification algorithm on unseen data. The study sample was divided into five equivalent subsets or “folds,” with random partitioning stratified by the number of patients reporting “antidepressant discontinuation” to ensure a roughly equal distribution across all folds. The ML classifiers were then trained and validated on different combinations of these folds. This process ensured a robust estimate of model performance while also allowing for hyperparameter tuning.

#### 2.6.6. Metrics of evaluation

To assess the efficacy of the machine learning classifiers, we used various standard performance metrics, including the Area Under the Curve-Receiver Operating Characteristic (AUC-ROC), the Area Under the Precision-Recall Curve (AUC-PRC), Cumulative Gain Curve, Sensitivity, Specificity, Positive Predictive Value (PPV), and F1-score (the harmonic mean between precision and recall).

AUC-ROC measures the trade-off between the True Positive Rate (Sensitivity) and the False Positive Rate (1-Specificity), and it is invariant to class distribution. On the other hand, AUC-RP demonstrates the trade-off between TPR and Precision. As our goal is to identify patients at risk of antidepressant discontinuation, thereby maximizing sensitivity and specificity, we ranked the ML models based on AUC-ROC. We calculated the mean and standard deviation (std) for each ML classifier over the nested 5-fold cross-validations.

#### 2.6.7. Hyperparameter optimization in machine learning models

In our machine learning models, each classifier's performance was optimized by tuning various parameters, which were chosen based on their significance in model building and prediction.

A detailed list of the specific values tested for each of these parameters in each machine learning classifier is presented in a table in Appendix F. This table provides a summary of the different parameter settings explored during the hyperparameter tuning phase.

#### 2.6.8. Statistical analysis

All data analyses were executed utilizing Python programming language's Scikit-learn, Seaborn, Scikit-plot, and Pylab. We presented descriptive statistics of qualitative perceived risk factors, intake duration, and ADR expressions as means (standard deviation) for continuous variables, and as counts (percentages) for binary/categorical variables. For each ML model, we provided the mean and standard deviation of the performance metrics, as assessed over the nested 5-fold cross-validations. Moreover, for the highest performing ML model, we highlighted the significance of the top variables to facilitate understanding of the predictors in the risk identification model.

## 3. Results

### 3.1. Sample characteristics

Our dataset, sourced from the forum “askapatient.com,” contained posts from 891 patients, with 432 related to SSRI antidepressants (Lexapro and Zoloft), and 459 linked to SNRI antidepressants (Effexor XR and Cymbalta). The posts were recorded from February 2001 through to September 2016. As Table 2 shows, patient demographics vary by antidepressant class. On average, patients reported using antidepressants for ~18 months, with the SSRIs taken for an average of 19 months and SNRIs for around 17 months. For SSRIs, the duration varied between 1 day and 16 years, while for SNRIs, the range was from 1 day to 20 years.

**Table 2:** Sample characteristics by antidepressants class.

**Drug class**	**Sample size (%)**	**Gender**	**Average age**	**Duration of intake**
SSRI	432 (48%)	F 310 (72%)	Avg. 35	Avg. 19 months
		M 118 (28%)	Med. 34	Med. 5 month
			Range 14–73	Range 1 day−16 years
SNRI	459 (52%)	F 359 (79%)	Avg. 38	Avg. 17 months
		M 94 (21%)	Med. 37	Med. 5 months
			Range 14–83	Range 1 day−20 years
Total	891	F 669 (75%)	Avg. 37	Avg. 18 months
		M 212 (25%)	Med. 35	Med. 5 months
			Range 14–83	Range 1 day−20 years

### 3.2. Risk factors for antidepressant discontinuation

Table 3 details the descriptive analysis of the risk factors associated with adherence and discontinuation of antidepressants. These factors are divided into what are generally considered as positive factors for adherence (i.e., expected to be associated with a lower discontinuation rate) and negative factors (i.e., anticipated to correlate with discontinuation of antidepressants). As anticipated, the absence of ADR and low perceived distress from ADR were rarely reported among patients discontinuing their medication. Interestingly, however, 41.5% of SSRI and 33.33% of SNRI patients, who discontinued their medication, found their antidepressants effective.

**Table 3:** A descriptive statistic of identified risk factors.

**Factors**	**SSRI discontinuation (*N* = 106)**	**SNRI discontinuation (*N* = 126)**
**Positive factors** ***N*** **(%)**		
ADR absence	4 (3.77%)	10 (7.93%)
Effectiveness	44 (41.5%)	42 (33.33%)
Perceived distress from ADR-low	3 (2.83%)	7 (5.55%)
**Negative factors** ***N*** **(%)**		
ADR presence	102 (96.22%)	116 (92.06%)
Perceived distress from ADR-high	82 (77.35%)	83 (65.87%)
Perceived distress from ADR-medium	19 (17.92%)	31 (24.6%)
Partial effectiveness^*^	21 (19.81%)	16 (12.69%)
Ineffectiveness	16 (15.09%)	33 (26.19%)
Patient's lack of knowledge about antidepressant	8 (7.54%)	16 (12.69%)
Patient forgetfulness to intake antidepressant	3 (2.83%)	5 (3.96%)

* Patients who also reported period of antidepressant ineffectiveness.

Negative factors such as the presence of ADR and high levels of perceived distress from ADR, showed the highest association with antidepressant discontinuation. A large majority of patients who discontinued (*n* = 232) mentioned at least one ADR in their posts, with 96.22% of these patients on SSRIs, and 92.06% on SNRIs. Notably, the percentage of patients discontinuing the antidepressant decreased significantly when they reported a medium level of distress, compared to a high level. For instance, 96.22% of SSRI patients who reported high distress discontinued the antidepressant, but this rate dropped to 17.92% for those reporting medium distress levels.

### 3.3. The most common ADRs reported for SSRIs and SNRIs

**Figure 2:**
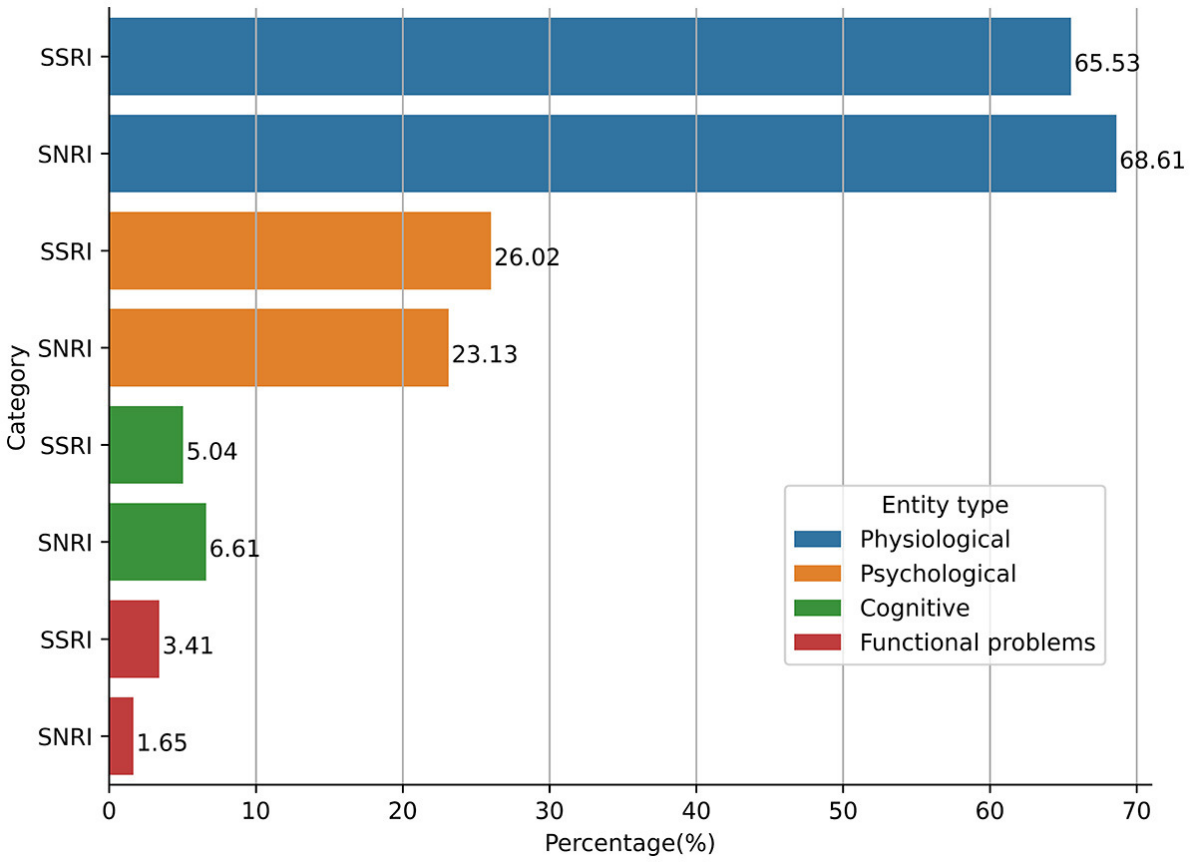
Top five ADRs reported for SSRI and SNRI antidepressants.

The type of ADRs reported by patients who discontinued SSRIs and SNRIs (*N* = 232) were analyzed by category of ADRs (i.e., physiological, psychological, cognitive, and functional problems). Figure 2 shows the top five reported ADRs for each category for SSRI and SNRI classes separately. The most common reported ADRs for SSRIs were weight gain (23.58%) and lack of libido (16.98%), and sleeplessness. Anxiety and foggy feeling in the head are the most commonly reported psychological and cognitive symptoms, respectively (see Appendix G for more details). Moreover, 2.83% of patients using SSRIs also reported experiencing Emergency Department visits. Among patients using SNRIs, the most commonly reported ADRs were physiological. Similar to the SSRIs, sleepiness, fatigue, and lack of libido are among the top five reported physiological ADRs (see Appendix H for more details).

### 3.4. Most informative ADRs identified by the JMIM method

**Figure 3:**
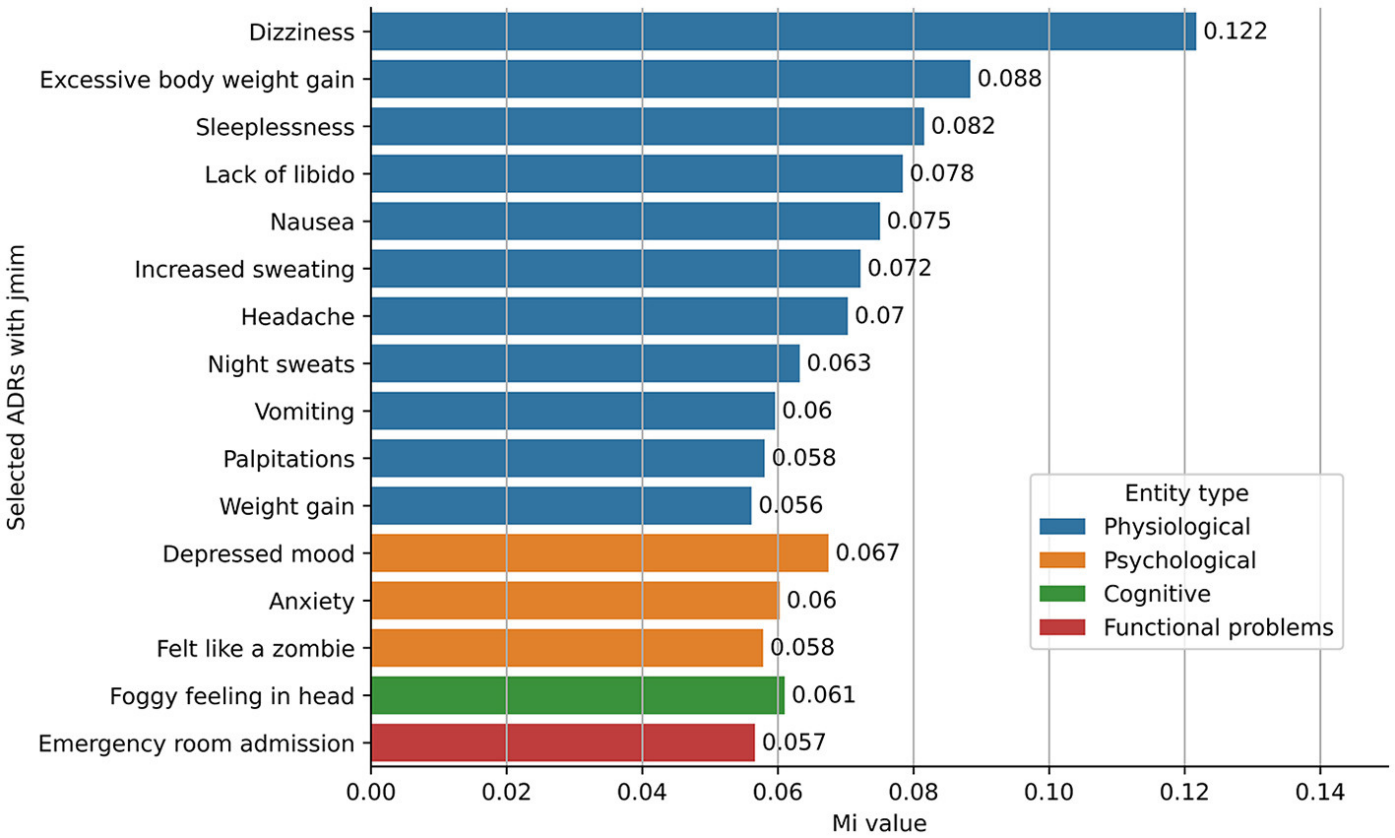
Most informative ADRs identified using JMIM feature selection method.

Figure 3 illustrates the 16 most informative features as identified by the JMIM method. Of the 673 normalized ADRs (refer to Section 2.5), the top five informative ADRs of antidepressant discontinuation included dizziness, excessive weight gain, sleeplessness, lack of libido, and nausea. Predominantly, ADRs fell within the physiological category (11 out of 16 informative ADRs), followed by the psychological category (three out of 16 informative ADRs). The cognitive category contributed “foggy feeling in the head” as the only representative among the most significant ADRs, whereas from the functional problems category, the only informative ADR was emergency room admission associated with antidepressant discontinuation. These 16 informative ADRs, along with demographic information and other risk factors from the PsyRisk corpus, were utilized to enhance the generalizability of the ML classifiers for the risk identification model.

### 3.5. Performance of ML models

We constructed various ML models utilizing predictor and outcome variables to anticipate patients with depression at risk of antidepressant discontinuation. Table 4 details the performance of these ML classifiers, employing the nested 5-fold cross-validation method. Notably, the Extra-Trees classifier demonstrated the highest AUC-ROC of 90.77 (89.00, 92.54). This is a significant 18% increase from the baseline Logistic Regression classifier, which had an AUC-ROC of 72.65 (69.63, 75.67). This improvement is primarily attributable to the Extra-Trees algorithm's structure, which employs the entire dataset for training the weak learners and selects random split points for tree growth, consequently reducing model variance and bias. Furthermore, this result implies a non-linear relationship between input and output variables. Overall, ensemble decision tree algorithms (with a mean AUC ranging from 88.46 to 90.77) outperformed the SVM method (with a mean AUC of 81.70). While SVM is frequently recognized as an optimal algorithm for smaller datasets with a high-dimensional feature space, our study's feature selection method led to the identification of 25 distinct features. This refined set of features subsequently enhanced the performance of ensemble decision tree algorithms, making them more effective than the SVM in this context. The Logistic Regression classifier demonstrated relatively lower performance (F1-score = 68.15 and AUC-ROC = 72.65), suggesting that the relationship between the risk factors and antidepressant discontinuation is not linear.

**Table 4:** Performance of ML algorithms.

**Algorithms**	**Precision**	**Recall**	**F1-score**	**Accuracy**	**AUC**
Extra-Trees	85.61 (82.41, 88.81)	**81.33** (77.35, 85.31)	**83.33** (80.75, 85.91)	**83.76** (81.37, 86.15)	**90.77** (89.00, 92.54)
Random Forest	**86.37** (82.6, 90.14)	76.78 (72.21, 81.35)	81.25 (77.42, 85.08)	82.32 (78.87, 85.77)	89.78 (87.76, 91.80)
XGBoost	85.26 (82.93, 87.59)	76.17 (73.98, 78.36)	80.44 (78.55, 82.33)	81.49 (79.76, 83.22)	88.80 (87.75, 89.85)
AdaBoost	83.38 (78.63, 88.13)	77.38 (73.58, 81.18)	80.13 (77.65, 82.61)	80.80 (78.25, 83.35)	88.46 (87.51, 89.41)
SVM	74.46 (73.72, 75.20)	76.48 (74.40, 78.56)	75.44 (74.56, 76.32)	75.11 (74.55, 75.67)	81.70 (80.58, 82.82)
Logistic Regression	66.36 (63.73, 68.99)	70.10 (68.02, 72.18)	68.15 (66.16, 70.14)	67.22 (64.88, 69.56)	72.65 (69.63, 75.67)

Bold values denote the highest computed score for each metric.

### 3.6. The most informative features identified by Extra-Trees and Random Forest ML models

**Figure 4:**
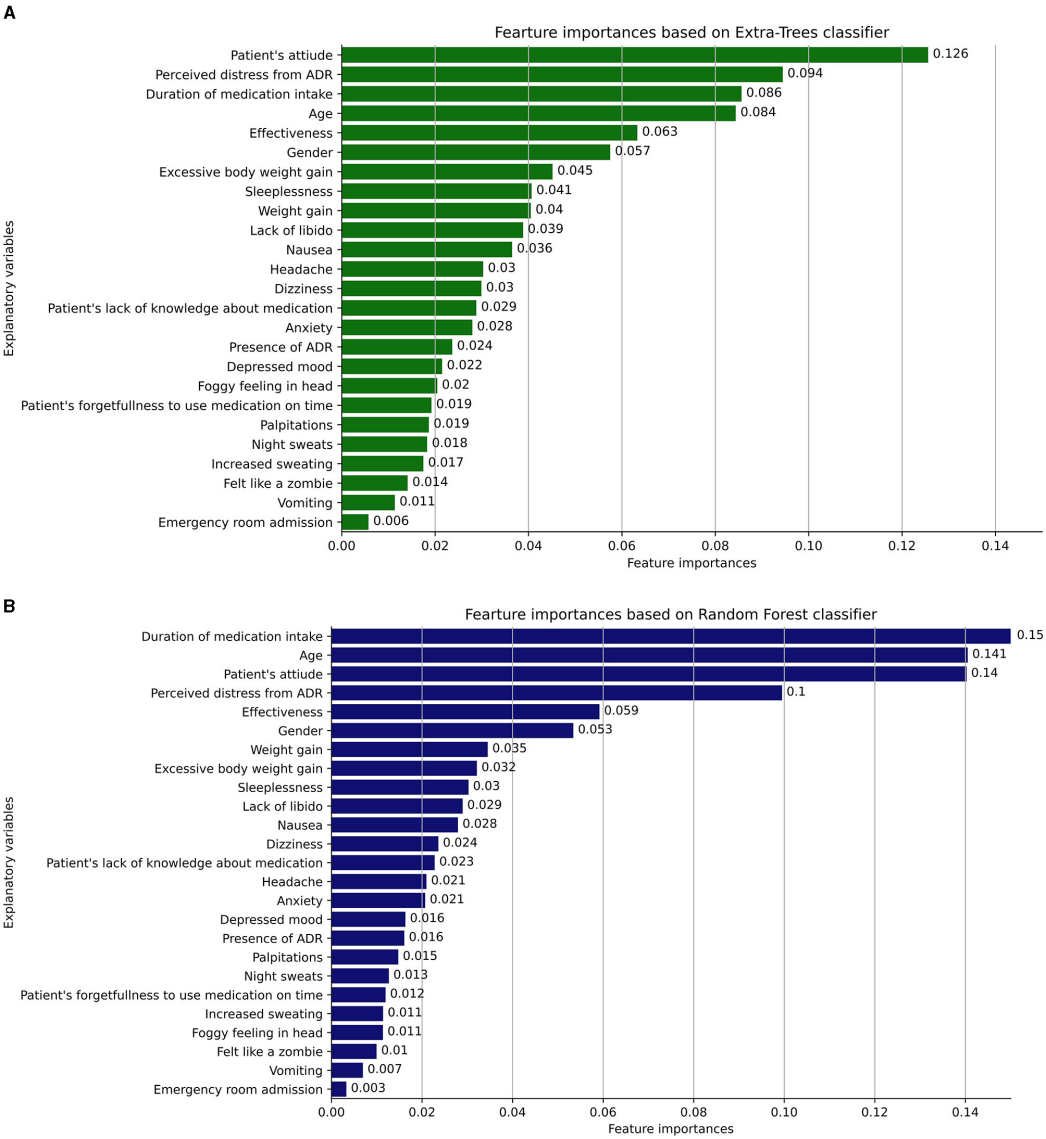
**(A, B)** The most informative features that influence patients' non-adherence behavior to antidepressants, as identified by the Extra-Trees and Random Forest classifiers, respectively.

Figures 4A, B illustrate the most informative features that influence patients' non-adherence behavior to antidepressants, as identified by the Extra-Trees and Random Forest classifiers, respectively. The Extra-Trees and Random Forest classifiers share several similarities in the ranking of explanatory variables related to ADRs and patient factors. Both models place high importance on variables such as “Duration of medication intake,” “Age,” “Patient's attitude,” and “Perceived distress from ADR.” These shared rankings indicate common insights into factors influencing antidepressant non-adherence behavior, reflecting a consensus on the key elements affecting non-adherence behavior in patients.

Despite these similarities, some differences emerge in the mid to lower rankings of importance between the two models. For example, variables like “Headache,” “Anxiety,” and “Depressed mood” are ranked differently, indicating variations in how these ADRs are weighed. A noticeable difference is the inclusion of “Excessive body weight gain” in the Extra-Trees classifier, absent in the Random Forest. These discrepancies highlight nuanced differences between the two models, suggesting a potential impact on the understanding and management of risk factors, and underlining the importance of model-specific consideration when interpreting the results.

### 3.7. Added value of each data component

**Figure 5:**
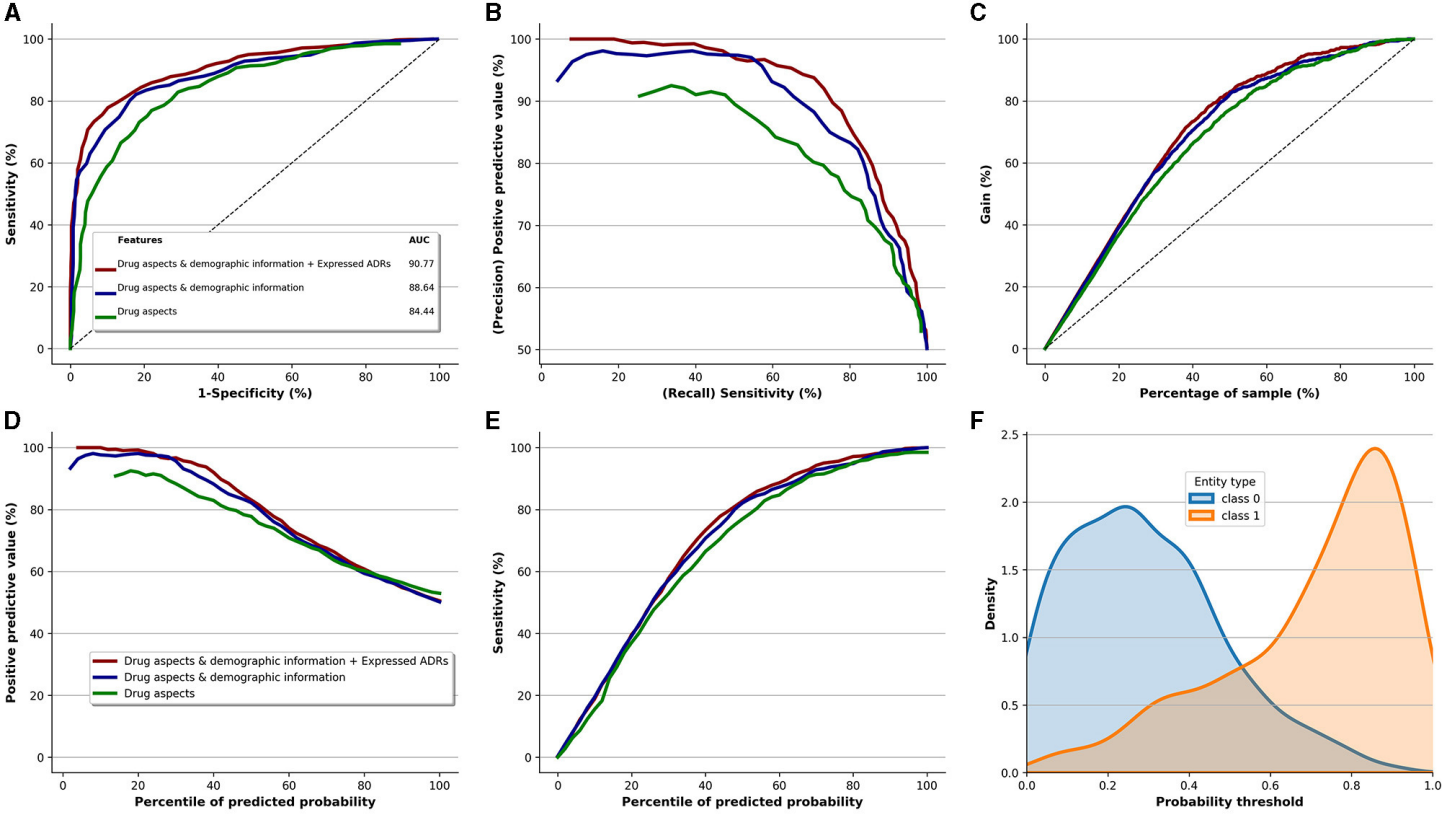
Performance of the Extra-Trees classifier (best performing model) and the added value of qualitative risk factors, demographic information, and type of ADRs. **(A)** ROC. **(B)** Precision vs. recall. **(C)** Cumulative gains curve. **(D)** Positive predictive value. **(E)** Sensitivity. **(F)** Prediction density.

Figure 5 displays the performance of the Extra-Trees classifier, our top-performing model, for identifying patients at risk of discontinuing their antidepressants. It also showcases the incremental value added by each data component (perceived quality, demographic information, and ADR expression). The base variables, derived from the perceived quality of the antidepressants, led to an AUC-ROC of 84.44. By incorporating demographic information with the base variables, we observed an AUC-ROC improvement of 4.20, yielding an AUC-ROC of 88.64. Furthermore, the integration of perceived ADRs with perceived quality and demographic information further boosted the AUC-ROC by 2.13, resulting in an AUC-ROC of 90.77. This underscores that variables indicating perceived quality of antidepressants are the most informative predictors of discontinuation. Additionally, it highlights the informational value of reported ADR types in predicting antidepressant discontinuation. A similar pattern of enhancement is evident for the Precision-Recall curve (Figure 5B), Positive Predictive Value curve (Figure 5D), and Sensitivity curve (Figure 5E).

Figure 5C depicts the incremental value brought by the three data components, relative to the sample size of the study. The gain curve demonstrates that if we target the top 40% of the entire population, which corresponds to 356 patients out of 891, this subset would comprise roughly 80% of the patients at risk of discontinuing antidepressants. That equates to ~186 patients (0.8 × 232 patients who discontinued medication). However, the perceived quality of antidepressants alone delivers about 70% gain for the same sample size, translating to around 162 patients (0.7 × 232).

## 4. Discussion

Non-adherence with treatment and abrupt discontinuation are common problems among patients using antidepressants. Such actions increase the risks of adverse mental health outcomes like depression relapse, low quality of life, and withdrawal symptoms, thereby posing a significant burden on the healthcare system. Currently, there exists a noticeable gap in data-based analysis tackling this issue, a gap that this study attempted to fill.

In addressing the medication discontinuation issue, we identified the need for a high-quality dataset to represent an online community perspective. Hence, we harnessed the richness of patient narratives available on social media, enabling a deep dive into patient-centric accounts. We adopted a unique method to annotate and curate the collected data, ensuring its quality and credibility. This methodological rigor allowed us to capture the multifaceted and nuanced aspects of antidepressant use, including adverse drug reactions and patient perceptions. Consequently, we constructed a robust, high-quality dataset that laid a solid foundation for our analysis.

With a high-quality annotated dataset of patients' self-reported experiences of antidepressants, we reported the various types of ADRs for four antidepressants from the SSRI and SNRI medications categories. The ADRs were broken down into physiological, psychological, cognitive, and functional categories. Factors like dizziness, excessive body weight gain, sleeplessness, lack of libido (sextual dysfunction), nausea more were among the top physiological ADRs reported. Past research corroborates our findings, specifically indicating that physiological ADRs, particularly sexual dysfunction, are associated with antidepressant non-adherence (Gregorian et al., 2002; De las Cuevas et al., 2014). Physiological ADRs can significantly impact a patient's experience with antidepressants and prompt discontinuation. The distress caused by these ADRs could outweigh the therapeutic benefits, prompting premature cessation of medication.

Beyond the descriptive summary of these adverse effects and risk factors, this study has also developed a series of machine learning algorithms to identify patients at risk of discontinuation. We tested the performance of different machine learning classifiers, including bagging and gradient boosting ensemble decision trees, SVM, and Logistic Regression. The ensemble decision trees displayed the highest performance in predicting patients at risk, particularly the Extra-Trees algorithm (F1-score = 83.33, AUC-ROC = 90.77), underlining the robustness of machine learning in this context.

The significance of this study lies in its novel use of self-reported data to identify discontinuation risk. The findings underscore that patients' self-reported experiences with pharmacological treatment contain vital clues about risk factors leading to medication non-adherence. Such knowledge can equip prescribers with the knowledge to tailor patient education in depression therapy and increase healthcare provider awareness of self-reported information that might lead a patient to discontinue antidepressant use, allowing them to act preemptively to avoid poor adherence.

In light of these findings, integration of these risk factors with existing clinically generated data from Electronic Health Records (EHR) can enhance patient-provider communication (Sirey et al., 2017), streamline reporting procedures, and refine dosage adjustments (Marasine and Sankhi, 2021). Moving forward, our research endeavor encompasses the development of a Natural Language Processing (NLP) system. This system aims to automatically extract potential risk factors from patients' self-reported experiences with antidepressants. With this implementation, we anticipate a comprehensive understanding of the patient's progress during the treatment, recognizing risk factors that may hinder this progress, and devising targeted interventions to amplify the effectiveness of treatment while mitigating adverse outcomes.

### 4.1. Limitations

The interpretation of this study's results should be done while considering several limitations.

Firstly, the demographic distribution of our SSRI and SNRI samples, primarily comprising females (~75% for both groups) with a median age range in the 30's, may influence the generalizability of the results. However, a 2021 review of factors affecting medication non-adherence noted that most non-adherent patients were under 40 years of age, aligning with our sample demographics (Henssler et al., 2019).

Secondly, our dataset, comprised of 891 antidepressant reviews divided into 6,009 sentences, is significant for a study requiring in-depth manual annotations like ours. However, compared to typical datasets in other domains, ours may appear relatively small. This constraint is primarily due to the intensive labor involved in a thorough examination by annotators, inherently limiting the volume of manageable data. It's important to note that the limited dataset size could influence the generalizability of the machine learning models' outcome. Further, the limited scope of our dataset might not fully capture some important risk factors. To attain a comprehensive understanding and identification of a wider range of risk factors, future research should consider the integration of larger datasets from diverse platforms and clinical settings.

Thirdly, our study's data source was the single healthcare forum, “askapatient.com.” While this platform provided valuable patient insights, it may not entirely encapsulate the diverse perspectives available across a variety of healthcare forums. Additionally, there's a potential selection bias in the forum's user base, as it often attracts individuals who are frequent internet users and particularly proactive about their health. This subset may not represent the broader patient population accurately, potentially biasing our data and skewing the predictions of our machine learning models. For example, the overrepresentation of these health-conscious patients could lead our models to overestimate medication adherence and health knowledge in the general population, potentially increasing false-negative rates. Also, essential risk factors among less health-conscious individuals may be underrepresented, limiting our models' accuracy and generalizability. As a future direction, integrating more diverse data sources and carefully considering these potential biases when interpreting predictive outcomes could enhance the scope and accuracy of our findings.

Fourthly, while previous studies have demonstrated the reliability of patient self-reported experiences in healthcare forums, the risk of fake or inaccurate reporting cannot be entirely discounted. The self-reporting nature of our data source introduces the possibility of misinterpreted or over/under-reported experiences.

Fifthly, despite the rigorous double coding of risk factors in all sentences of antidepressant reviews, there is a chance of misinterpretation by annotators leading to the assignment of the risk factor to an incorrect sentence.

Sixthly, it's possible that the reported adverse drug reactions (ADRs) could be attributable to other medications or herbal treatments the patient was taking concurrently with the antidepressant. Similarly, patient interpretation of the effectiveness of the antidepressants and the reported ADRs might be subjective and vary from one patient to another.

Lastly, the scope of our study was limited to SSRI and SNRI classes of antidepressants, potentially restricting the generalizability of our findings to other antidepressant classes, such as Tricyclic Antidepressants (TCAs).

Despite these limitations, our study represents a novel contribution to understanding patient experiences with antidepressants and providing a data-driven approach to predicting medication discontinuation risk.

## 5. Conclusion

Adherence to antidepressant therapy is a crucial factor in achieving successful treatment response and remission, yet non-adherence remains a significant challenge in clinical practice. This study introduced the PsyRisk corpus, a collection of identified risk factors linked to the abrupt discontinuation of antidepressants, derived from patients' self-reported experiences on online platforms. This study utilized social media data, which may allow for the inclusion of more diverse patients from different geographic areas, potentially addressing a significant need for broader research across varied regions. This study utilized social media data, which may allow for the inclusion of more diverse patients from different geographic areas, potentially addressing a significant need for broader research across varied regions. The analysis highlighted the importance of patient perceived antidepressant effectiveness and patient distress from ADRs in influencing patient antidepressant non-adherence, with physiological ADRs identified as particularly relevant. By utilizing the risk factors extracted from the PsyRisk and PsyTAR corpora (a collection of patients' expressions of ADRs related to antidepressants), a promising risk identification algorithm was developed. This tool has shown potential in identifying characteristics indicative of patients at risk of abruptly discontinuing their antidepressant medication. Timely identification of these patients can enable personalized interventions, such as tailored patient education on antidepressant medications, improved patient-provider communication, and shared decision-making regarding medication adjustments. This may potentially mitigate patient distress, improve treatment satisfaction, and ultimately, enhancing patient adherence and safety.

## Data availability statement

The datasets and codes presented in this study can be found online via the following link: https://github.com/zonour97/Antidepressant_discontinuation/tree/main.

## Ethics statement

The studies involving humans were approved by University of Wisconsin-Milwaukee. The studies were conducted in accordance with the local legislation and institutional requirements. Written informed consent for participation was not required from the participants or the participants' legal guardians/next of kin in accordance with the national legislation and institutional requirements.

## Author contributions

AZ: data analysis and preparing the manuscript. CE: literature review, data analysis, and manuscript preparation. AF: data collection and reviewing the manuscript. MK, DF, ZZ, and HM: data annotation and reviewing the manuscript. YY, AD, AT, SB, and TO: manuscript preparation. MT, HK, KF, PF, MR, and TP: designing the methodology and reviewing the manuscript. MZ: designing the methodology, manuscript preparation, data annotation, and data analysis. All authors contributed to the article and approved the submitted version.
