# Supplementary material for: A risk identification model for detection of patients at risk of antidepressant discontinuation
Source: Front Artif Intell. 2023 Aug 24;6:1229609. doi: 10.3389/frai.2023.1229609 (PMC10484003; doi:10.3389/frai.2023.1229609)

**Supplementary documents**

**Appendix A. structure of data collected by the healthcare forum “askapatient.com”**

| **Rating (Satisfaction)** | **Reason** | **Side-effect** | **Comment** | **Gender** | **Age** | **Duration (Duration of intake)** | **Date** |
| --- | --- | --- | --- | --- | --- | --- | --- |
| 3 | fibromyalgia/depression | Nausea, diarrhea, upset stomach, dry mouth, sleepiness | I have only been on 20mg for 6 days and have the extreme runs. Upset stomach and low appetite. Pain is less though and I am less anxious and depressed. | M | 42 | 4 days | 2010-5-15 |

**Appendix B. Details of creating sample size of the study**

We collected information for four antidepressant medications, namely Zoloft (Generic name: Sertraline), Lexapro (Escitalopram) from SSRI class, and Effexor XR (venlafaxine) and Cymbalta (duloxetine) from SNRI class. According to the National Institutes of Health (NIH) MedlinePlus, these antidepressants are among the most prevalent ones prescribed in the United States.[23] We used the following sample size formula[32] to calculate the size of population for each group of patients:

$$\text{Sample Size}= \frac{\frac{Z^{2}\times P(1-P)}{E^{2}}}{1+\left( \frac{Z^{2}\times P(1-P)}{E^{2}N} \right)}$$

We set Z as the confidence interval of 90%, *E as* the margin of error of 5%, and *P* as the standard deviation of 0.5 to ensure we obtained a sufficient sample size reflecting the patients’ population in the forum. N is the total number of the patient population who reported their experiences in the forum. The calculated sample size for Zoloft, Lexapro, Cymbalta, and Effexor XR were 213, 219, 231, and 228, respectively.

**Appendix C. Joint Mutual Information Maximization (JMIM) method**

“To identify significant ADRs associated with ADRs, we used a Joint Mutual Information Maximization[33] (JMIM) method. In information theory, the mutual information (MI) of two random variables is the amount of information obtained about one random variable (X) by observing the other random variable (Y). This can be quantified as the reduction in entropy of one random variable (Y) given another variable (X) as follows:

$$I\left( X;Y \right)=E\left( Y \right)-E\left( Y|X \right)$$

Where $E\left( Y \right)$is the entropy of $E\left( Y \right)$. For any discrete variable such Y = (y_1_, y_2_, …, y_N_), E(Y) is defined as:

$$E\left( Y \right)= -\sum_{i=1}^{N} p\left( y_{i} \right)\log(p(y_{i}))$$

$E\left( Y|X \right)$ is conditional entropy. The conditional entropy is the amount of uncertainty left in variable Y when variable X is introduced. So, it is less than or equal to the entropy of both variables. Conditional entropy is formulated as follows:

$$E\left( Y|X \right)= -\sum_{j=1}^{M} \sum_{i=1}^{N} p\left( x_{i}, y_{j} \right)\log(p(y_{j}|x_{i}))$$

The information gain method is the simplest feature selection method built on Mutual Information (MI). Simplicity and computational efficiency are the main advantages of this method. However, like statistical-based methods such as Chi-square, it assumes features are conditionally independent of one another. To tackle this problem, JMIM method attempted to take into account the potential dependency among feature set F = {f_1_, f_2_, …, f_N_} by selecting a subset of feature S = {s_1_, s_2_, …, s_k_} with dimension of K where $K\left( number of features in S \right)<N(number of featues in F)$ and $S\subseteq F$; While minimizing the redundancy of information among selected features and maximizing the joint mutual information among a subset of S and outcome class label Y. Mathematically,

$$f_{JMIM}=\arg\max_{f_{i} \in F-S} (\min_{f_{s} \in S} (I(f_{i}, f_{s};Y)))$$

The biggest advantage of the JMIM method compared to feature selection methods from the class of wrapper and embedding is its generalizability of selected features, which can improve the stability and generalizability of the ML models on an unseen dataset.

**Appendix D. Development and evaluation ML models for predicting antidepressant effectiveness**

**Input variables:** The input variables for predicting antidepressant effectiveness included: ADR presence, perceived distress from ADR, patient lack of knowledge about antidepressant, patient forgetfulness to intake antidepressant, duration of intake, satisfaction with the antidepressant, and patient’s age and gender.

**Outcome variable:** The outcome variable is the binary variable of antidepressant effectiveness (effectiveness versus ineffectiveness)

**Machine learning classifiers:**

We used different discriminative machine learning (ML) algorithms, including: Logistic Regression as the baseline algorithm, bootstrap aggregation (bagging), and gradient boosting ensemble decision trees as non-parametric ML methods with the ability to generate a large number of decision trees (weak learners), and support of a vector machine (SVM) as a parametric ML algorithm with the ability to use linear and non-linear kernels.

We used two popular Bagging algorithms, Random Forest and Extremely Randomized Trees (Extra-Trees). For the gradient boosting decision tree methods, we used Adaptive Boosting (AdaBoost) and extreme Gradient boosting (XGBoost), two popular methods from this boosting category. See more explanation of the ML algorithms in **Appendix E.**

**Using five-fold cross-validation to evaluate the performance of the ML classifiers**

Since our dataset is relatively small, we used stratified cross-validation to provide a better picture of the generalizability of the risk identification algorithm on unseen data. Following this approach, we partitioned the study sample into five equal subsects (“folds”), with the random partitioning stratified by the number of patients who reported medication effectiveness versus ineffectiveness. The ML classifiers were trained using the data in the four folds (80% of data) and tested on the 5th fold. This procedure was repeated five times until each fold was used for testing.

**Result:** Table 1 presents the performance of ML classifiers used to measure the effectiveness of the antidepressant. We used the Extra-Trees classifier to impute missing values for effectiveness in the patients’ antidepressants reviews. Extra-Trees had the highest performance compared to other ensemble decision tree algorithms and SVM. The AUC-ROC = 92.04 with F1-score = 80.22 indicates that the ML model we developed for measuring antidepressant effectiveness is a suitable method and, therefore, can be used for the imputation of antidepressant effectiveness in the dataset.

**The performance of ML classifiers in measuring antidepressants effectiveness**

| **Algorithms** | **Precision** | **Recall** | **F1-score** | **Accuracy** | **AUC-ROC** |
| --- | --- | --- | --- | --- | --- |
| Extra-Trees | 80.57 ± 1.49 | 80.36 ± 1.27 | 80.22 ± 1.14 | 80.36 ± 1.27 | 92.04 ± 0.51 |
| Random Forest | 80.39 ± 1.75 | 80.11 ± 1.74 | 79.54 ± 2.21 | 80.03 ± 1.96 | 92.24 ± 0.51 |
| XGBoost | 78.98 ± 1.82 | 78.96 ± 1.71 | 78.70 ± 1.71 | 78.96 ± 1.71 | 91.76 ± 0.67 |
| AdaBoost | 71.68 ± 1.86 | 71.28 ± 2.23 | 70.71 ± 2.65 | 71.28 ± 2.23 | 83.42 ± 1.51 |
| SVM | 67.91 ± 2.00 | 68.56 ± 1.70 | 67.93 ± 1.93 | 68.56 ± 1.70 | 85.56 ± 0.90 |
| Logistic Regression | 64.14 ± 3.08 | 65.42 ± 2.61 | 64.26 ± 3.00 | 65.42 ± 2.61 | 83.04 ± 1.47 |

**Appendix E. Machine learning algorithms used for this study**

We used different discriminative machine learning (ML) algorithms, including (i) logistic regression as baseline algorithm, (ii) bootstrap aggregation[34] (bagging), and Gradient Boosting[35] ensemble decision trees as non-parametric ML methods with abilities to generate a large number of decision trees (weak learners) and aggregating their outcomes to make a better prediction, and (iii) support vector machine (SVM) as a parametric ML algorithm that allows transformation of features using linear and non-linear kernels.

In Bagging methods, weak learners are trained independently and in parallel on the entire or subsets of the training sample and weighted equally for computing the final outcome.[34] We used two popular algorithms, Random Forest and Extremely Randomized Trees[36] (Extra Trees) from Bagging methods, for this study. On the other hand, in Gradient Boosting methods, weak learners are generated sequentially, taking into account the error of the previous decision tree algorithm, and are weighted according to their performance for computing the final outcomes.[35] We used Adaptive Boosting[37] (AdaBoost) and extreme gradient boosting (XGBoost)[38], two popular methods from this boosting category.

Logistic Regression uses a logistic function, an optimization function, to estimate the coefficient of each input variable (predictor) in predicting the outcome variables. The LR performance is often used as a baseline binary ML classification model because of its simplicity and efficiency in implementation.[39]

Bagging methods are ensemble decision tree algorithms in which weak learners are trained independently and in parallel on the entire or subsets of the training sample and weighted equally for computing the final outcome.[34] We used two popular algorithms, Random Forest and Extremely Randomized Trees[36] (Extra Trees) from Bagging methods, for this study. Random Forest generates each weak learner from a bootstrap sample (a resampling technique used to estimate statistics on a population by sampling a dataset with replacement), while the Extra Trees algorithm fits each weak decision tree on the entire original learning sample. Both algorithms use a subset of features randomly selected at each split point (nodes) for growing the weak learner trees. However, unlike Random Forest, which uses a greedy algorithm to choose an optimal split point, the Extra Trees algorithm selects the split point entirely at random. For both algorithms, outcomes of the weak trees are aggregated using the majority votes in classification problems to yield the final prediction. Extra Trees uses explicit randomization of the cut-point and the entire original learning sample rather than bootstrap replicas, reducing variance and bias significantly more than the Random Forest with a weaker randomization scheme.[36]

Gradient Boosting methods are ensemble decision tree algorithms in which weak learners are generated sequentially, taking into account the error of the previous decision tree algorithm, and are weighted according to their performance for computing the final outcomes.[35] We used Adaptive Boosting[37] (AdaBoost) and extreme Gradient boosting (XGBoost)[38], two popular methods from this boosting category. AdaBoost creates an ensemble of weak learners iteratively by modifying the weights of misclassified data in each iteration. Weak learners with misclassified outcomes receive larger weights; therefore, they have a higher probability of appearing in the training sample than the succeeding weak learners. Unlike AdaBoost, which changes the distribution of sample distribution for training weak learners, XGBoost uses a gradient descent algorithm (an algorithm for minimizing a loss function) to reduce the number of errors as weak learners are incrementally on the remaining residual errors of a strong learner. Therefore, it does not alter the sample distribution. Both algorithms use optimization algorithms to select features for growing trees and split points of the nodes. However, unlike XGBoost, which uses optimization parameters to compute the depth of the weak learners, AdaBoost creates weak learners with a single split, called decision stumps. Additionally, XGBoost uses regularization parameters to reduce the regression tree function’s complexity and the model’s variance bias (the bias-variance trade-off).

SVMs belong to the general category of kernel methods.[40] A kernel method is an algorithm whose dependence on data is only limited to the inner products of data vectors. Therefore, the inner products can be replaced by a kernel function that computes the inner product in high-dimensional data. The advantage of this approach is, first, the ability to generate non-linear boundaries using algorithms for linear classifiers. Second, use of the kernel function allows the user to apply a classifier to data without fixed-dimensional vector space representation. SVM was built on five basic concepts[41]: (i) the separating hyperplane for dividing the data concerning their outcome label; (ii) the maximum-margin hyperplane for maximizing the generalizability of the SVM; (iii) the soft margin, which uses the regularization parameter that controls the trade-off between maximization the margin and minimizing the misclassification error; (iv) kernel function, a function that uses similarity measures for transform data and reducing the vector dimensions. The kernel function reduces the data complexity and improves the separability of data, which is particularly useful in high-dimensional feature space. Studies showed that optimizing SVM parameters reduces variation and bias in prediction tasks.[41], [42]

**Appendix F: Hyperparameter tuning in machine learning classifiers**

For the Extra-Trees classifier, we tuned the 'min_samples_split', 'max_features', and 'n_estimators'. The 'min_samples_split' parameter determines the minimum number of samples required to split an internal node. The 'max_features' parameter indicates the number of features to consider when looking for the best split. The 'n_estimators' parameter controls the number of trees in the forest.

For the Random Forest classifier, we adjusted the 'n_estimators', 'max_features', and 'criterion'. The 'n_estimators' controls the number of trees in the forest. The 'max_features' parameter sets the maximum number of features considered for splitting a node, while the 'criterion' parameter defines the function to measure the quality of a split.

In the case of the XGBoost model, the parameters 'min_child_weight', 'gamma', 'subsample', 'colsample_bytree', and 'max_depth' were tuned. 'Min_child_weight' gives the minimum sum of instance weight needed in a child, 'gamma' controls the minimum loss reduction required to make a further partition, and 'subsample' and 'colsample_bytree' control the subsample ratio of the training instance and the subsample ratio of columns when constructing each tree. 'Max_depth' sets the maximum depth of a tree.

For the AdaBoost classifier, we optimized the 'n_estimators' and 'learning_rate'. 'N_estimators' sets the maximum number of estimators at which boosting is terminated, while 'learning_rate' shrinks the contribution of each classifier.

In the Support Vector Machine (SVM) classifier, we adjusted 'kernel', 'degree', and 'gamma'. The 'kernel' specifies the kernel type to be used in the algorithm, 'degree' is the degree of the polynomial kernel function ('poly'), and 'gamma' defines the kernel coefficient.

Lastly, for the Logistic Regression classifier, we tuned the 'penalty' and 'C'. The 'penalty' parameter specifies the norm used in the penalization, while 'C' is the inverse of regularization strength, smaller values specifying stronger regularization.

**Range of parameter values tested for each machine learning classifier**

| **ML algorithms** | **Parameters** |
| --- | --- |
| Extra-Trees | params = {  'min_samples_split': [2, 5, 15],  'max_features': [1, 5, 10],  'n_estimators': [50, 500, 1000, 5000]  } |
| Random Forest | params = {  'n_estimators': [10, 100, 500, 1000],  'max_features': [2, 4, 6, 8],  'criterion': ['gini', 'entropy']  } |
| XGBoost | params = {  'min_child_weight': [1, 5, 10],  'gamma': [0.5, 1, 1.5, 2, 5],  'subsample': [1.0, 0.8, 0.6],  'colsample_bytree': [0.6, 0.8, 1.0],  'max_depth': [3, 4, 5]  } |
| AdaBoost | Params = {  'n_estimators': [500, 1000, 2000, 5000],  'learning_rate': [.001,0.01,.1]  } |
| SVM | params = {  'kernel': ['poly', 'rbf', 'linear'],  'degree': [2, 4, 8],  'gamma': ['scale', 'auto']  } |
| Logistic Regression | params = {  'penalty': ['l1', 'l2', 'elasticnet'],  'C': [0.1, 1, 10]  } |

**Appendix G: The most common reported ADRs for patients who discontinued SSRIs**


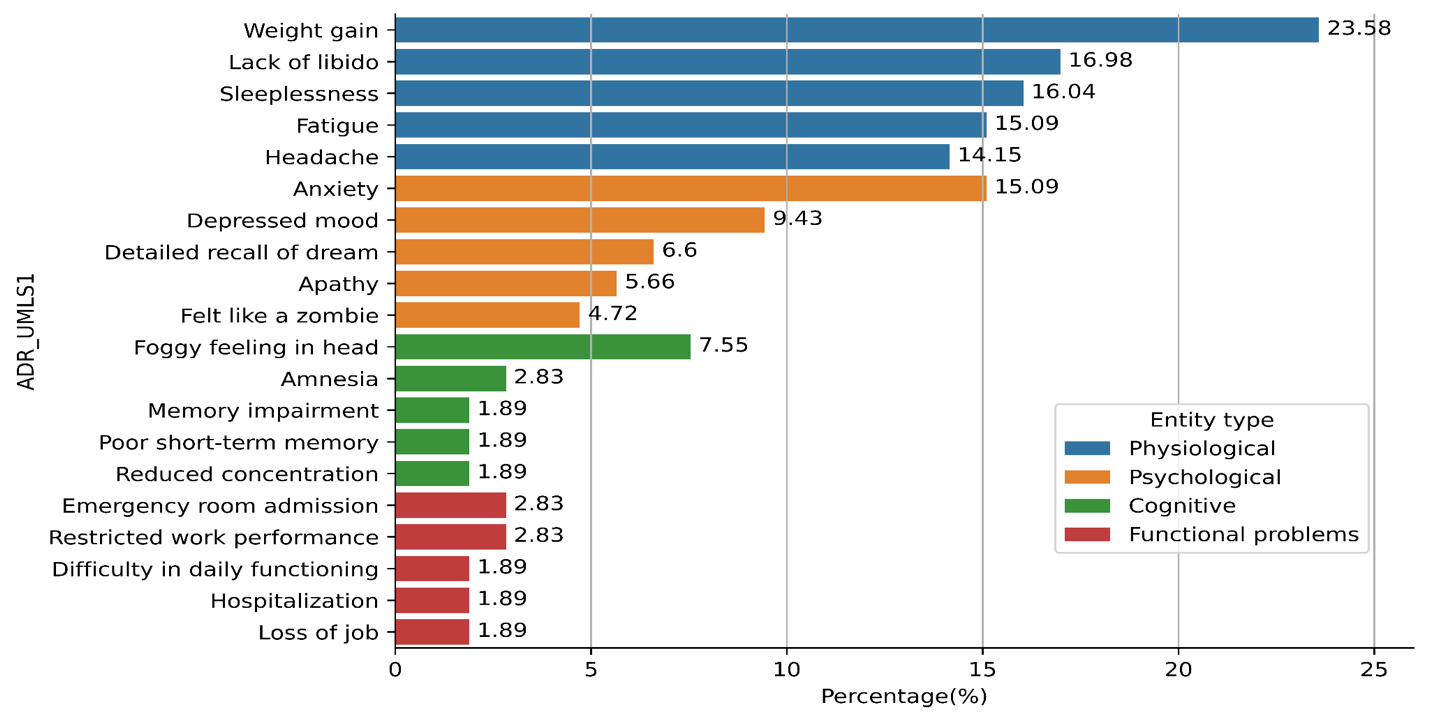


**Appendix H: The most common reported ADRs for patients who discontinued SNRIs**


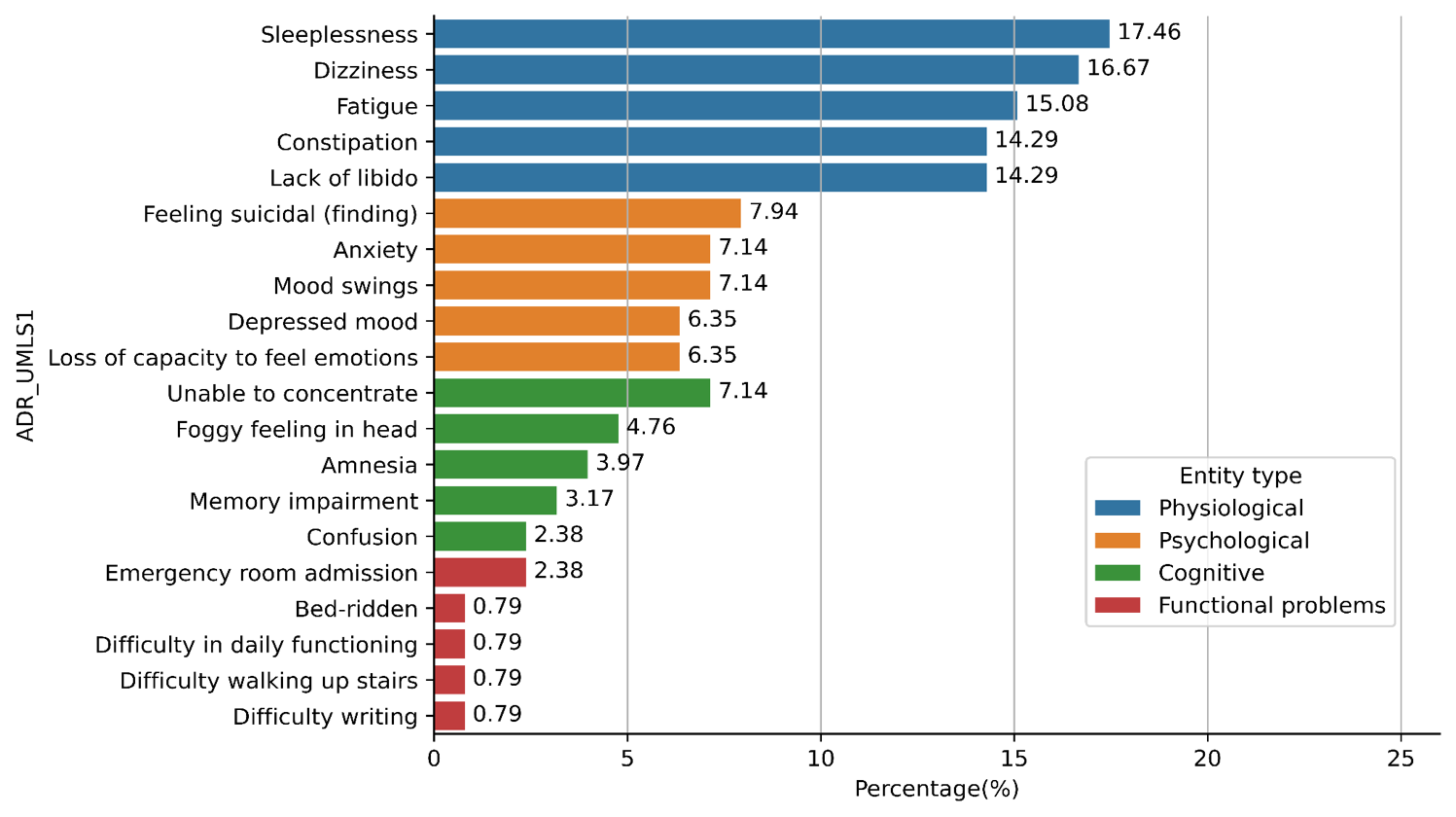

Supplement: Supplementary file 1 [file Data_Sheet_1.docx]
